# Foreign Correspondence

**Published:** 1869-11

**Authors:** 


### Foreign Correspondence.

---

BERLIN, *September 8th, 1869.*

DEAR EXAMINER:—When in Wiesbaden, I was interested in calling at the laboratory of Prof. Fresenius, having used his book of Qualitative Analysis in America, which, with his Quantitative Analysis, is one of the best books in the hands of the student. I believe the laboratory is unconnected with any other institution. It accommodates about 50 students, and is well furnished with apparatus, but not so convenient as some of our similar laboratories at home. The Professor lectures several times each week. The course occupies about two years, at an expense of $100 a year. There is some instruction also given in pharmacology, mineralogy, and organic chemistry. The school is especially adapted for analytical chemists, and is but little frequented by medical students.

At Bonn, the lecture-room had closed, and no clinics were being given. I visited the surgical wards of the Catholic Hospital, which had many interesting cases, but were in the most slovenly, neglected condition of any wards I have yet seen. The building was erected for a castle, and is in a measure unfit for its present use. One of the cases was an infant about two weeks old, which had been operated on for artificial anus.

Before the operation, at about the third day, the child was icteric and melanotic. A considerable quantity of meconium and fæces were discharged at the completion of the incision. The child rapidly attained a normal color, and when I saw it, seemed in a hopeful and comfortable condition. Bonn has about 20,000 population, and, of course, does not afford very extensive hospitals. About 1700 out-patients had visited the medical clinic this year, up to August 28th, and about an equal number the surgical clinic. Prof. Veit is an able lecturer on obstetrics and diseases of women, and has written a book of some value on the latter subject. Prof. Rindfleisch is one of the attractions of the university, being one of the most reputed authors on pathological anatomy in the country. Bonn has about 190 medical students, 60 of whom attend the clinics.

Göttingen has a neat and orderly kept hospital, with about 130 patients, among whom were twelve or more with venereal disease.

The Anatomy Museum contains something over 4000 specimens, among which is a collection of over 300 cranii, of different races of men.

There were several specimens injected with quicksilver, beautifully illustrating the lymphatic system.

Prof. Schweigger gives four lectures and four clinics on the eye each week, and two hours on the use of ophthalmoscope. Prof. Baum gives instruction in surgical operations, four hours a week, and a daily clinic. Lectures on general surgery five times a week, by Prof. Lohmeyer.

Dr. Marmé gives lectures on electro-therapie, with practical application of the induced and constant currents, twice each week. The medical department of the university, however, is best resorted to for anatomy, physiology, and chemistry, which are provided with numerous and able instructors, such as Profs. Henle, Krämer, Krause, Meissner, Wöhler, Fittig, Von Uslar, Boedeker, and others. The medical students number 150.

The Berlin medical school vies in exellency with those of Paris, London, and Vienna; and it is impossible to form any correct idea of its advantages at this season of the year, when lectures and courses have ceased, and many of the professors away for recuperation and pleasure. It would be somewhat surprising if Berlin, with about 700,000 population, and a celebrated university, should leave anything undone that could be done, to render every possible facility to the medical student to acquire knowledge. Virchow and Rokitansky, Frerichs and Oppolzer, Gräfe and Arlt, Martin and Braun, Von Langenbeck and Billroth, as representatives of the Berlin and Vienna schools, are all men of profound learning and vast experience; and, practically, they can be said to scarcely excel each other in their particular departments. Many Prussians go to Vienna, some Austrians come to Berlin. But as I often have occasion to remark to German students, Americans do not resort to Europe to hear their medical professors, as in many departments we have their equals or superiors in America, but rather to see and examine their patients; and whatever city gives the greatest freedom in this respect will be the more attractive to us.

The largest hospital of Berlin, Charité, has an average list of about 1360 patients, and is a model of cleanliness and order. In this are most of the public and many of the private courses given. There are several other hospitals in the city, accommodating from 50 to 400 patients, some of which are accessible to students. To the German student who has five years to his course, or the specialist, I presume Berlin furnishes as good opportunities, and, perhaps, in some, better than Vienna; but for most Americans who wish to carry on several subjects at the same time, he can undoubtedly accomplish his ends better in Vienna, where the patients are concentrated in one building.

Much more attention is given to skin disease and venereal in Vienna than here. The death of Prof. Böhm, and continued feebleness of Prof. Gräfe, leave the eye department a little unsettled here. Chemistry, anatomy, and physiology are more advantageously studied here.

Vienna has about 1200 students, Berlin 500. Those who have studied at both places, so far as I have learned, give testimony in favor of Vienna. Yours truly, F.

